# Immune microenvironment, homologous recombination deficiency, and therapeutic response to neoadjuvant chemotherapy in triple-negative breast cancer: Japan Breast Cancer Research Group (JBCRG)22 TR

**DOI:** 10.1186/s12916-022-02332-1

**Published:** 2022-04-25

**Authors:** Takayuki Ueno, Shigehisa Kitano, Norikazu Masuda, Daiki Ikarashi, Makiko Yamashita, Tomohiro Chiba, Takayuki Kadoya, Hiroko Bando, Takashi Yamanaka, Shoichiro Ohtani, Shigenori Nagai, Takahiro Nakayama, Masato Takahashi, Shigehira Saji, Kenjiro Aogi, Ravi Velaga, Kosuke Kawaguchi, Satoshi Morita, Hironori Haga, Shinji Ohno, Masakazu Toi

**Affiliations:** 1 Breast Surgical Oncology, The Cancer Institute Hospital of JFCR, 3-8-31, Ariake, Koto-ku, Tokyo, 135-8550 Japan; 2 Division of Cancer Genomic Medicine Development, Advanced Medical Development Center, The Cancer Institute Hospital of JFCR, Tokyo, Japan; 3 Division of Cancer Immunotherapy Development, Advanced Medical Development Center, The Cancer Institute Hospital of JFCR, Tokyo, Japan; 4 Department of Breast and Endocrine Surgery, Nagoya University Graduate School of Medicine, Nagoya, Japan; 5 Division of Pathology, The Cancer Institute Hospital of JFCR, Tokyo, Japan; 6 Department of Breast Surgery, Hiroshima University Hospital, Hiroshima University, Hiroshima, Japan; 7 Breast and Endocrine Surgery, Faculty of Medicine, University of Tsukuba, Ibaraki, Japan; 8 Department of Breast and Endocrine Surgery, Kanagawa Cancer Center, Yokohama, Japan; 9 Department of Breast Surgery, Hiroshima City Hiroshima Citizens Hospital, Hiroshima, Japan; 10 Division of Breast Oncology, Saitama Cancer Center, Saitama, Japan; 11 Department of Breast and Endocrine Surgery, Osaka International Cancer Institute, Osako, Japan; 12 Department of Breast Surgery, NHO Hokkaido Cancer Center, Sapporo, Japan; 13 Department of Medical Oncology, Fukushima Medical University Hospital, Fukushima, Japan; 14 Department of Breast Oncology, National Hospital Organization Shikoku Cancer Center, Matsuyama, Ehime Japan; 15 Department of Breast Surgery, Kyoto University Hospital, Kyoto, Japan; 16 Department of Biomedical Statistics and Bioinformatics, Kyoto University Graduate School of Medicine, Kyoto, Japan; 17 Department of Diagnostic Pathology, Kyoto University Hospital, Kyoto, Japan; 18 Breast Oncology Center, The Cancer Institute Hospital of JFCR, Tokyo, Japan; 19 Department of Breast Surgery, Kyoto University Graduate School of Medicine, Kyoto, Japan

**Keywords:** Triple-negative breast cancer, Homologous recombination deficiency (HRD), *BRCA1/2*, Immune microenvironment, Neoadjuvant chemotherapy, Platinum, Eribulin

## Abstract

**Background:**

Triple-negative breast cancer (TNBC) is a biologically diverse disease, with characteristics such as homologous recombination deficiency (HRD), gene mutation, and immune reactions. Japan Breast Cancer Research Group 22 is a multicenter trial examining TNBC’s response to neoadjuvant chemotherapy (NAC) according to the HRD status. This translational research investigated the clinical significance of the immune microenvironment of TNBC in association with HRD, tumor *BRCA1/2* (tBRCA1/2) mutation, and response to NAC.

**Methods:**

Patients aged below 65 years with high HRD or germline *BRCA1/*2 (gBRCA1/2) mutation randomly received paclitaxel + carboplatin (group A1) or eribulin + carboplatin (A2), followed by anthracycline. Patients aged below 65 years with low HRD or those aged 65 years or older without gBRCA1/2 mutation randomly received eribulin + cyclophosphamide (B1) or eribulin + capecitabine (B2); nonresponders to the first four cycles of the therapy received anthracycline. A pathological complete response (pCR) was defined as the absence of residual cancer cells in the tissues. Pretreatment biopsy specimens were stained by multiplexed fluorescent immunohistochemistry using antibodies against CD3, CD4, CD8, Foxp3, CD204, and pan-cytokeratin. Immune cells with specific phenotypes were counted per mm^2^ in cancer cell nests (intratumor) and stromal regions. The immune cell densities were compared with clinicopathological and genetic factors including tumor response.

**Results:**

This study analyzed 66 samples. T1 tumors had a significantly higher density of intratumoral CD8^+^ T cells than T2 or larger tumors. The tBRCA1/2 mutation or HRD status was not associated with the density of any immune cell. The density of intratumoral and stromal CD4^+^ T cells was higher in patients showing pCR than in those without pCR. In a multivariate analysis, intratumoral and stromal CD4^+^ T cell density significantly predicted pCR independent of age, chemotherapy dose, HRD status, and treatment groups (*P* = 0.009 and 0.0057, respectively). In a subgroup analysis, the predictive value of intratumoral and stromal CD4^+^ T cell density persisted in the platinum-containing chemotherapy group (A1+A2) but not in the non-platinum-containing group (B1+B2).

**Conclusions:**

Intratumoral and stromal CD4^+^ T cell density was an independent predictor of pCR in patients with TNBC. A larger study is warranted to confirm the results.

**Trial registration:**

UMIN000023162

**Supplementary Information:**

The online version contains supplementary material available at 10.1186/s12916-022-02332-1.

## Background

Triple-negative breast cancer (TNBC) is a subset of breast cancer without the expression of estrogen receptor (ER) and progesterone receptor (PgR) and without the overexpression or gene amplification of human epidermal growth factor receptor 2 (HER2) [1]. TNBC still has no specific target for treatment; thus, the current standard systemic therapy for early-stage TNBC is conventional chemotherapy, which includes anthracycline and taxane. However, the prognosis of patients with TNBC is unfavorable compared with other subtypes of breast cancer and the clinical outcome still has to be further improved, and new treatment strategies are needed [1, 2].

TNBC is a biologically diverse disease. Its biological characteristics include homologous recombination deficiency (HRD), gene mutation, and immune reactions [2–4]. Around half of TNBC has been reported to have HRD due to mutations or promotor hypermethylation of relevant genes such as *BRCA1/2*, *PALB2*, and *RAD51C* [5–7]. The assay to score HRD using cancer tissues has been developed based on loss of heterozygosity, telomeric allelic imbalance, and large-scale state transitions [8]. Several studies examined the association between HRD score and response to chemotherapy, in particular platinum agents, but the results were inconsistent [9–12].

TNBC shows a relatively high mutation burden compared with other breast cancer subtypes, and *TP53* is the most frequently mutated gene in TNBC [13–15]. In general, a higher mutation burden is considered to lead to more neoantigen production. Indeed, the association between higher mutation burden and greater immune reaction has been reported in a variety of cancers [16]. However, in TNBC, increased immune reactions have been reported to be associated with lower mutation burden and lower clonal heterogeneity, suggesting an immune editing effect where cancer progression by clonal expansion is suppressed by immune surveillance [15, 17].

Tumor-infiltrating lymphocytes (TILs) are associated with prognosis; they can predict TNBC response to neoadjuvant chemotherapy (NAC) [18–25]. The International Immuno-Oncology Biomarker Working Group published recommendations for the standardized assessment of TILs in breast cancer tissues [26, 27]. Several international guidelines, such as the European Society of Medical Oncology Guidelines, have included TILs as a prognostic biomarker [28]. However, the subsets of lymphocytes that contribute the most to the prognostic and predictive values of TILs for TNBC remain unclear.

Japan Breast Cancer Research Group 22 (JBCRG22) trial is a multicenter trial that examined response to NAC according to the HRD status in patients with TNBC and showed good pCR rates of 65% and 45% by weekly paclitaxel + carboplatin and eribulin + carboplatin, respectively [29]. This translational research aimed to investigate the clinical significance of the immune microenvironment of TNBC in association with HRD, tumor *BRCA1/2* (tBRCA1/2) mutation, and response to NAC in order to give further insights in TNBC biology including tumor microenvironment for the improvement of treatment strategies of TNBC.

## Methods

### JBCRG22 study

The study design, endpoints, and results of JBCRG22 have been reported previously [29]. Briefly, patients aged below 65 years with TNBC showing a high HRD status (HRD score ≧ 42) (Myriad Genetics, Inc., Salt Lake City, Utah) [8] or germline *BRCA1/2* (gBRCA1/2) mutation randomly received 4 cycles of either weekly paclitaxel 80mg/m^2^ on days 1, 8, and 15 + carboplatin AUC6 on day 1 of a 21-day cycle (group A1) or eribulin 1.4mg/m^2^ on days 1 and 8 + carboplatin AUC6 on day 1 of a 21-day cycle (group A2), followed by an anthracycline-containing regimen (5-fluorouracil–epirubicin–cyclophosphamide, FEC or doxorubicin–cyclophosphamide, AC) every 21 days for 4 cycles. Patients aged below 65 years with TNBC showing a low HRD status (HRD score < 42) or those aged 65 years or older without gBRCA1/2 mutation were randomly assigned to 6 cycles of either eribulin 1.4mg/m^2^ on days 1 and 8 + cyclophosphamide 600mg/m^2^ on day 1 of a 21-day cycle (group B1) or eribulin 1.4mg/m^2^ on days 1 and 8 + capecitabine 2000mg/m^2^ b.i.d. for 14 days of a 21-day cycle (group B2); nonresponders to the first 4 cycles of the therapy received an anthracycline-containing regimen (FEC or AC) every 21 days for 4 cycles. The major exclusion criteria included having bilateral breast cancer, multiple cancers other than breast cancer, axillary lymph node dissection before the study treatment, and severe uncontrolled systemic diseases [29].

All study participants provided written informed consent. The institutional review board approved the research protocol, which conformed to the Declaration of Helsinki.

### Pathological assessment of treatment response

The centralized pathologic review committee, as well as each participating institution, assessed the pathological response. The absence of residual cancer cells in the tissues indicated a pathological complete response (pCR).

### Multiplex fluorescent immunohistochemistry

As previously described [30], pretreatment biopsy specimens in formalin-fixed paraffin-embedded blocks were stained by multiplexed fluorescent immunohistochemistry with an Opal IHC kit (AKOYA Biosciences, CA, USA) using antibodies against CD3 (clone SP7; Abcam, Tokyo, Japan), CD4 (4B12; Leica Microsystems, Tokyo, Japan), CD8 (4B11; Leica Microsystems, Tokyo, Japan), Foxp3 (D608R; Cell Signaling Technology, Danvers, MA, USA), CD204 (SRA-E5; TransGenic, Kobe, Japan), and pan-cytokeratin (AE1/AE3, Dako). Briefly, a whole slide was scanned using an automated imaging system (Vectra ver. 3.0, AKOYA Biosciences). After being stained by hematoxylin and eosin, the tissue slides were used to annotate the tumoral and stromal fields according to the International Immuno-Oncology Biomarker Working Group’s recommendation [27]. The whole specimens were captured, with an average of 20 areas at ×200 magnification. Using an image-analyzing software (InForm, AKOYA Biosciences), we segmented tumor tissues into cancer cell nests and stromal regions and identified immune cells with specific phenotypes (Additional file 1: Fig. S1). Before the final evaluation, manual training sessions for tissue segmentation and phenotype recognition were conducted, followed by automatic machine learning for the algorithm. An analytic software program (Spotfire, TIBCO software, CA, USA) counted the infiltrating immune cells with specific phenotypes per mm^2^ in cancer cell nests (intratumor) and stromal regions (stroma).

### Statistical analysis

We used the Mann–Whitney test for comparing two groups, the Kruskal–Wallis test for comparing more than two groups, and the *χ*^2^-test for comparing pCR rates between groups. For multivariate analyses, logistic regression analysis was performed. The doses of paclitaxel in group A1 and eribulin in groups A2, B1, and B2 were used for the analyses. All statistical data were analyzed using the JMP version 13.2.1 (SAS Institute, Inc., Cary, NC, USA). All *P* values were two-sided, and a *P* value of less than 0.05 was considered statistically significant. All graphs were produced using the GraphPad Prism version 8.4.3 (GraphPad Software, San Diego, CA, USA) and the JMP version 13.2.1.

## Results

### Background characteristics of patients

A total of 66 TNBC samples from the JBCRG22 study were available and analyzed in this study. Table 1 summarizes the background characteristics of these 66 patients. The age of patients in group A (A1 or A2) was lower than that in group B (B1 or B2) because treatment groups A and B have different entry criteria including age.

**Table 1:** Patient background characteristics and pathological response to neoadjuvant chemotherapy

Treatment group		Total	A1	A2	B1	B2
Patient number		66	15	18	17	16
Age, years	Median	54	44	47	58	56
	min.–max.	26–70	31–64	26–63	35–70	41–70
T	T1c	13	5	3	2	3
	T2	48	9	13	14	12
	T3	5	1	2	1	1
N	N0	39	7	11	11	10
	N1	27	8	7	6	6
Histological grade (B and R)	1	2	1	0	0	1
	2	17	2	4	5	6
	3	44	11	14	11	8
	Unknown	3	1	0	1	1
Ki67 labeling index, %	Median	57.7	55	64.2	51.6	50.4
	min.–max.	16.2–90	20.2–90	36.4–89.6	20–89	16.2–82
HRD	Low	18	0	0	9	9
	High	33	15	18	0	0
	Unknown	15	0	0	8	7
tBRCA1/2 mutation	*BRCA1* mutation	6	3	3	0	0
	*BRCA2* mutation	6	3	3	0	0
	No mutation	43	9	12	12	10
	Unknown	11	0	0	5	6
pCR	Yes	23	10	8	2	3
	No	43	5	10	15	13
	%	34.8	66.7	44.4	11.8	18.8

*A1* group taking paclitaxel + carboplatin, *A2* group taking eribulin + carboplatin, *B1* eribulin + cyclophosphamide, *B2* eribulin + capecitabine, *HRD* homologous recombination deficiency, *tBRCA* tumor *BRCA*, *max.* maximum, *min.* minimum, *pCR* pathological complete response

### Immune cells in cancer tissues in association with background characteristics

**Fig. 1:**
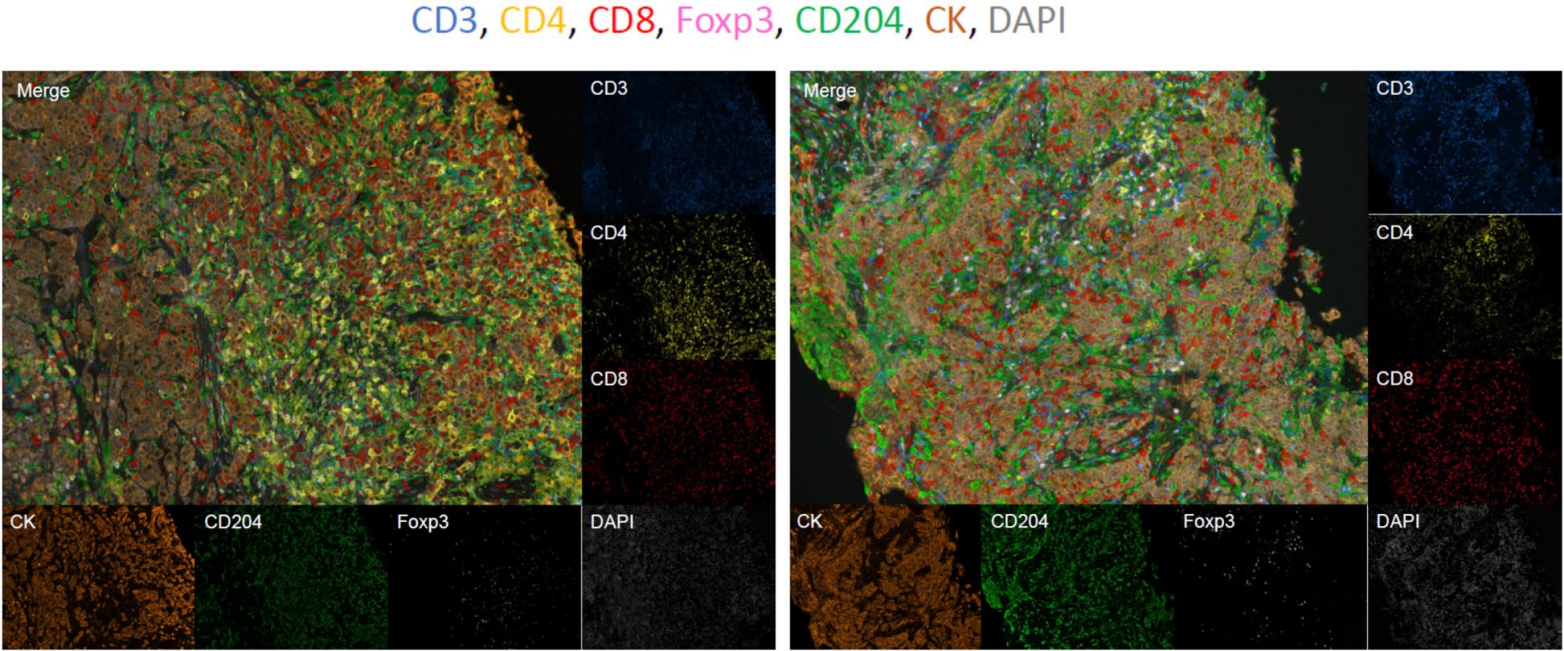
Representative images of multiplex fluorescent immunohistochemistry. Multiplex fluorescent immunohistochemistry was performed using antibodies against CD3 (blue), CD4 (yellow), CD8 (red), Foxp3 (pink), CD204 (green), and pan-cytokeratin (brown)

**Fig. 2:**
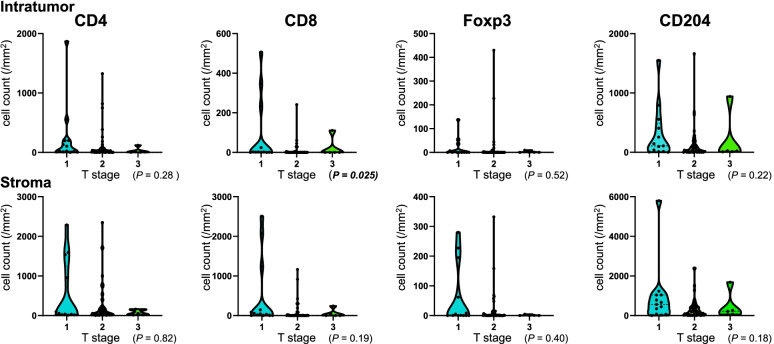
Immune cell density according to T stage. The vertical axis indicates cell count/mm^2^ and the horizontal axis indicates tumor T stage. Statistically significant *P* values are shown in bold italics. The numbers of patients are as follows: T1, *N* = 13; T2, *N* = 48; and T3, *N* = 5. The density of intratumoral CD8^+^ T cells was higher in T1 tumors than in T2 or T3 tumors (*P* = 0.025)

The densities of CD3^+^CD4^+^ cells (CD4^+^ T cells), CD3^+^CD8^+^ cells (CD8^+^ T cells), CD4^+^Foxp3^+^ cells (Treg cells), and CD204^+^ cells were compared with the patients’ background tumor characteristics (Fig. 1). The density of intratumoral CD8^+^ T cells was associated with T stage (*P* = 0.025, Fig. 2), and it was highest in T1 tumor (≤2 cm). Conversely, nodal status, histological grade, and Ki67 labeling index were not associated with the density of any immune cell type.

### Tumor BRCA1/2 mutation and HRD status and immune cells

**Fig. 3:**
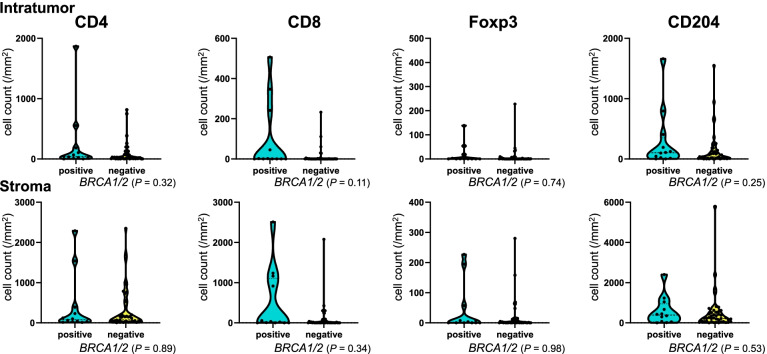
Immune cell density according to tumor *BRCA1/2* mutation status. The vertical axis indicates cell count/mm^2^, and the horizontal axis indicates the tumor *BRCA1/2* mutation status. The numbers of patients are as follows: positive, *N* = 12; negative, *N* = 43. Immune cell density showed no association with tumor *BRCA1/2* mutation status

**Fig. 4:**
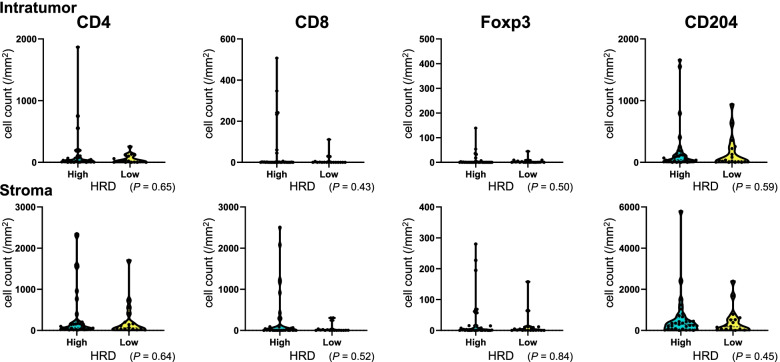
Immune cell density according to the HRD status. The vertical axis indicates cell count/mm^2^, and the horizontal axis indicates the tumor HRD status. The numbers of patients are as follows: HRD high, *N* = 33; HRD low, *N* = 18. Immune cell density showed no association with the HRD status

Patients with tBRCA1/2 mutation had a relatively high density of intratumoral and stromal CD8^+^ T cells, but no statistical significance was observed (Fig. 3). The HRD status was not associated with the density of any immune cells (Fig. 4).

### Treatment response and immune cells

**Fig. 5:**
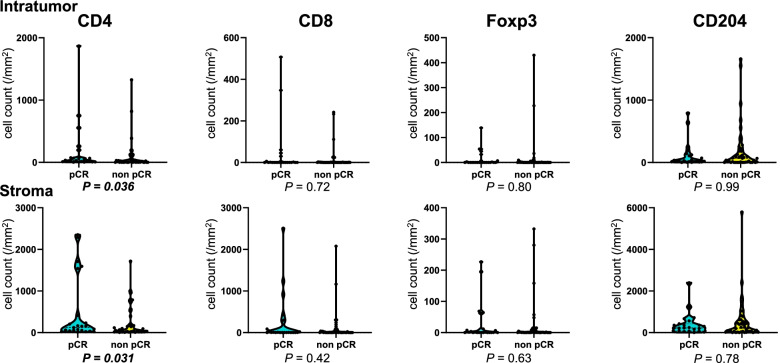
Immune cell density according to the pathological response. The vertical axis indicates cell count/mm^2^, and the horizontal axis indicates the pathological response. Statistically significant *P* values are shown in bold italics. The numbers of patients are as follows: pCR, *N* = 23; non-pCR, *N* = 43. Patients with pCR to neoadjuvant chemotherapy showed a higher density of intratumoral and stromal CD4^+^ T cells than those with non-pCR (*P* = 0.036 and 0.031, respectively).

Patients with pCR to NAC showed a higher density of intratumoral and stromal CD4^+^ T cells than those with non-pCR in the whole population (*P* = 0.036 and 0.031, respectively; Fig. 5). The multivariate analysis revealed that the density of intratumoral and stromal CD4^+^ T cells significantly predicted pCR independent of age, dose, HRD status, and treatment groups (*P* = 0.009 and 0.0057, respectively; Table 2).

**Table 2:** Multivariate analysis for pCR

	Intratumoral CD4			Stromal CD4		
	***HR***	95% ***CI***	***P*** value	***HR***	95% ***CI***	***P*** value
**Age**	0.93	0.853–1.011	0.072	0.9	0.821–0.996	*0.024*
**Dose**	1.073	1.005–1.147	*0.0073*	1.059	0.994–1.13	*0.032*
**HRD**	0.92	0.848–1.005	0.053	0.92	0.841–1.007	0.058
**Treatment**			*0.035*			*0.026*
**A1**	Reference			Reference		
**A2**	0.48			0.35		
**B1**	2.61×10^−11^			1.92×10^−11^		
**B2**	0.016			0.013		
**Intratumoral CD4**	1.008	0.997–1.019	***0.009***			
**Stromal CD4**				1.0023	1.0002–1.0044	***0.0057***

*A1* group taking paclitaxel + carboplatin, *A2* group taking eribulin + carboplatin, *B1* eribulin + cyclophosphamide, *B2* eribulin + capecitabine, *CI* confidence interval, *HR* hazard ratio, *Dose* dose of paclitaxel in group A1, eribulin in groups A2, B1 and B2, *HRD* homologous recombination deficiency, *pCR* pathological complete response

Statistically significant *P* values are shown in italics

Subgroup analyses of patients who received platinum-containing chemotherapy (groups A1+A2) and those who received non-platinum-containing chemotherapy (groups B1+B2) were performed. The HRD status was excluded from the analysis because all patients in the platinum-containing chemotherapy group had tumors with a high HRD status (Table 1). Consistent with the whole population, both intratumoral and stromal CD4^+^ T cell densities independently predicted pCR in the platinum-containing chemotherapy group (*P* = 0.018 and 0.022, respectively, Table 3), but not in the non-platinum-containing chemotherapy group (*P* = 0.38 and 0.73, respectively; Table 3). As an exploratory analysis, the predictive value of the density of each immune cell type for pCR was assessed in each treatment group, which showed no significant association of any immune cell type with pCR (Additional file 1: Fig. S2 A to D).

**Table 3:** Multivariate analysis for pCR according to treatment groups

Platinum-containing chemotherapy (groups A1 + A2)						
	***HR***	**95%** ***CI***	***P*** **value**	***HR***	**95%** ***CI***	***P*** **value**
**Age**	0.89	0.809–0.980	*0.0066*	0.88	0.797–0.973	*0.0038*
**Dose**	1.041	0.970–1.117	0.25	1.029	0.963–1.099	0.38
**Treatment**			0.32			0.19
**A1**	Reference			Reference		
**A2**	0.39			0.29		
**Intratumoral CD4**	1.009	0.996–1.022	***0.018***			
**Stromal CD4**				1.002	1.000–1.004	***0.022***
Non-platinum-containing chemotherapy (groups B1 + B2)						
	***HR***	**95%** ***CI***	***P*** **value**	***HR***	**95%** ***CI***	***P*** **value**
**Age**	1.02	0.893–1.169	0.75	1.009	0.883–1.153	0.89
**Dose**	1.091	0.975–1.222	*0.022*	1.096	0.968–1.241	*0.029*
**Treatment**			0.42			0.70
**B1**	Reference			Reference		
**B2**	2.47			1.573		
**Intratumoral CD4**	0.998	0.994–1.003	0.38			
**Stromal CD4**				1.0003	0.998–1.002	0.73

*A1* group taking paclitaxel + carboplatin, *A2* group taking eribulin + carboplatin, *B1* eribulin + cyclophosphamide, *B2* eribulin + capecitabine, *CI* confidence interval, *HR* hazard ratio, *Dose* dose of paclitaxel in group A1, eribulin in groups A2, B1 and B2

Statistically significant *P* values are shown in italics

In the extended analyses of CD4^+^/CD8^+^, CD4^+^/Foxp3^+^, Foxp3^+^/CD4^+^, and CD8^+^/Foxp3^+^ ratios, the pathological response was not associated with any of these ratios.

We further performed exploratory analyses to examine whether pCR rates differ depending on the immune phenotypes [31]. We categorized the immune microenvironment into three groups based on the densities of immune cells, either CD4^+^ T cells or CD8^+^ T cells, in cancer cell nests and stromal regions: immune inflamed, high cell density in both cancer cell nests and stromal regions; immune excluded, low cell density in cancer cell nests and high cell density in stromal regions; and immune desert, low cell densities in both cancer cell nests and stromal regions (Additional file 1: Fig. S3 A to C). No significant differences in pCR rate were observed between different immune phenotypes for both CD4^+^ T cells and CD8^+^ T cells (*P* = 0.43 and 0.33, respectively; Additional file 1: Fig. S3 D).

## Discussion

This study demonstrated that intratumoral and stromal CD4^+^ T cell densities were independent predictive factors of pCR in patients with TNBC who received NAC, particularly platinum-containing chemotherapy. However, previous studies that examined the predictive values of TILs for pCR to neoadjuvant platinum-containing chemotherapy obtained inconsistent results. A study using samples from GeparSixto showed that TILs were associated with pCR in patients with TNBC who received platinum-containing NAC [20]. In contrast, a pooled analysis of five phase II studies of neoadjuvant platinum-based chemotherapy in TNBC failed to show the predictive values of intratumoral and stromal TILs for pCR [32]. Although such discrepancy cannot be clearly explained, differences in combined chemotherapeutic agents could be one reason. Another explanation would be that among TILs, different subsets of immune cells may have different clinical significance in TNBC. In patients who received NAC with anthracycline- and/or taxane-based chemotherapies, CD8^+^ TILs are associated with pCR [33, 34]. Our results indicate that of all the cells in cancer tissues, CD4^+^ T cells may predict pathological response to platinum-containing NAC in patients with HRD-high TNBC. Thus, different subsets of immune cells may have different clinical values. A detailed analysis of the subset of TILs will help elucidate the clinical utilities of tumor-associated immune cells, leading to improved treatment strategies for TNBC.

When examining the clinical significance of immune cell subsets, assessing how to define each immune cell subset is important. For example, CD4 may be expressed not only in T cells but also in a subpopulation of monocytes, causing a bias and discrepancies between studies [35]. Our studies used CD4 or CD8 markers together with CD3; thus, we could better identify CD4^+^ or CD8^+^ T cell subset. When comparing the results from different studies, knowing the definition of each immune subset is crucial.

Our study revealed that immune cells were not associated with either tBRCA1/2 mutation or HRD status, consistent with a pooled analysis that showed no association between intratumoral or stromal TIL density and either the HRD status or tumor *BRCA1/2* mutation status in TNBC [32]. Thus, TILs and the HRD status or tBRCA1/2 mutation status may have distinct clinical values. In a study with 414 Danish patients with breast cancer showing gBRCA1/2 mutation, patients with gBRCA1 mutation had a higher rate of CD4^+^ cells than those with gBRCA2 mutation [36]. However, the present study did not compare patients with and without gBRCA1/2 mutation and examined patients with tumor mutation but not those with germline mutation.

Immune editing is a concept depicting the immune cell status depending on the cancer progression stage [17, 37]. In the elimination phase, immune cells work actively to eliminate developing cancer cells, as reflected by immune cell accumulation. In the equilibrium phase, immunologic mechanisms prevent cancer growth. Then, in the escape phase, the immune cells no longer block tumor growth. In our study, T1 tumors had a higher density of intratumoral CD8^+^ T cells than T2 or larger tumors, which might be a reflection of immune editing. This result is similar to those studies showing that larger tumors had a lower density of immune cells than smaller tumors [33, 34].

This study has several limitations. One of the major limitations is a small sample size in each treatment arm. Considering that JBCRG22 examined four different regimens according to the HRD status, each arm had a small number of patients; thus, the result in each single arm needs to be interpreted with caution. It is of clinical importance to conduct a larger study to validate the results in this study. Another limitation is that survival analysis was not performed because of the short follow-up period. The prognostic value of each immune subset needs to be clarified in future studies with a longer follow-up. Lack of the analysis on different types of CD4^+^ T cells such as naive, central memory, effector memory, and effector in terms of response to NAC is another limitation. Furthermore, in addition to the previously known Th1 and Th2 types of CD4^+^ T cells, the existence of Th9, Th17, and Th22 types of cells has recently been reported, but their roles remain unclear. Because studies on the clinical significance of different types of CD4^+^ T cells will give further insights in the field of immune microenvironment of TNBC, it is of clinical value to conduct such studies in the future.

## Conclusions

The density of intratumoral and stromal CD4^+^ T cells was an independent predictor for pCR to NAC, especially platinum-containing chemotherapies, in patients with TNBC. Because the sample size is limited in this study, a larger study is required to confirm the results.

## Supplementary Information

**Additional file 1: Figure S1.** Image analysis. Representative image of immunofluorescence with the following markers: CD3 (blue), CD4 (yellow), CD8 (red), FoxP3 (pink), CD204 (green), and cytokeratin (brown) (A); tissue segmentation of the intratumoral (red) and stromal (green) areas (B); cell segmentation (C) and cell phenotyping (D) which merged with tissue segmentation (E): cancer cells (orange), CD4^+^ T cells (yellow), CD8^+^ T cells (red), CD204^+^ cells (green), other stromal cells (gray). **Figure S2.** Immune cell density according to pathological response in each treatment group: group A1 (A), group A2 (B), group B1 (C), group B2 (D). **Figure S3.** Immune phenotype and pCR. (A) Immune inflamed, high cell density in both cancer cell nests and stromal regions; immune excluded, low cell density in cancer cell nests and high cell density in stromal regions; immune desert, low cell densities in both cancer cell nests and stromal regions. (B) Immune phenotype for CD4^+^ T cells. (C) Immune phenotype for CD8^+^ T cells. (D) pCR rate according to immune phenotype for CD4^+^ T cells and CD8^+^ T cells.

## Data Availability

The datasets used and/or analyzed during the current study are available from the corresponding author on reasonable request.

## Abbreviations

CD4^+^ T cells: CD3^+^CD4^+^ cells
CD8^+^ T cells: CD3^+^CD8^+^ cells
ER: Estrogen receptor
gBRCA: Germline BRCA
HER2: Human epidermal growth factor receptor 2
HRD: Homologous recombination deficiency
NAC: Neoadjuvant chemotherapy
pCR: Pathological complete response
PgR: Progesterone receptor
tBRCAm: Tumor BRCA mutation
TIL: Tumor-infiltrating lymphocyte
TNBC: Triple-negative breast cancer
Treg cells: CD4^+^Foxp3^+^ cells
