# Supplementary material for: Immune microenvironment, homologous recombination deficiency, and therapeutic response to neoadjuvant chemotherapy in triple-negative breast cancer: Japan Breast Cancer Research Group (JBCRG)22 TR
Source: BMC Med. 2022 Apr 25;20:136. doi: 10.1186/s12916-022-02332-1 (PMC9036790; doi:10.1186/s12916-022-02332-1)
Supplement: Supplementary file 1 — Additional file 1: Figure S1. Image analysis. Representative image of immunofluorescence with the following markers: CD3 (blue), CD4 (yellow), CD8 (red), FoxP3 (pink), CD204 (green), and cytokeratin (brown) (A); tissue segmentation of the intratumoral (red) and stromal (green) areas (B); cell segmentation (C) and cell phenotyping (D) which merged with tissue segmentation (E): cancer cells (orange), CD4+ T cells (yellow), CD8+ T cells (red), CD204+ cells (green), other stromal cells (gray). Figure S2. Immune cell density according to pathological response in each treatment group: group A1 (A), group A2 (B), group B1 (C), group B2 (D). Figure S3. Immune phenotype and pCR. (A) Immune inflamed, high cell density in both cancer cell nests and stromal regions; immune excluded, low cell density in cancer cell nests and high cell density in stromal regions; immune desert, low cell densities in both cancer cell nests and stromal regions. (B) Immune phenotype for CD4+ T cells. (C) Immune phenotype for CD8+ T cells. (D) pCR rate according to immune phenotype for CD4+ T cells and CD8+ T cells. [file 12916_2022_2332_MOESM1_ESM.docx]

**Additional File 1 - Immune microenvironment, homologous recombination deficiency and therapeutic response to neoadjuvant chemotherapy in triple negative breast cancer: Japan Breast Cancer Research Group (JBCRG)22 TR**

Takayuki Ueno^1,2*^, Shigehisa Kitano^3^, Norikazu Masuda^4^, Daiki Ikarashi^3^, Makiko Yamashita^3^, Tomohiro Chiba^5^, Takayuki Kadoya^6^, Hiroko Bando^7^, Takashi Yamanaka^8^, Shoichiro Ohtani^9^, Shigenori Nagai^10^, Takahiro Nakayama^11^, Masato Takahashi^12^, Shigehira Saji^13^, Kenjiro Aogi^14^, Ravindranath M. Velaga^15^, Kosuke Kawaguchi^15^, Satoshi Morita^16^, Hironori Haga^17^, Shinji Ohno^18^, Masakazu Toi^19^

^1^Breast Surgical Oncology, ^2^Division of Cancer Genomic Medicine Development, Advanced Medical Development Center, The Cancer Institute Hospital of JFCR, Tokyo, Japan. ^3^Division of Cancer Immunotherapy Development, Advanced Medical Development Center, The Cancer Institute Hospital of JFCR, Tokyo, Japan. ^4^Department of Breast and Endocrine Surgery, Nagoya University Graduate School of Medicine, Nagoya, Japan. ^5^Division of Pathology, The Cancer Institute Hospital of JFCR, Tokyo, Japan. ^6^Department of Breast Surgery, Hiroshima University Hospital, Hiroshima University, Hiroshima, Japan. ^7^Breast and Endocrine Surgery, Faculty of Medicine, University of Tsukuba, Ibaraki, Japan. ^8^Department of Breast and Endocrine Surgery, Kanagawa Cancer Center, Yokohama, Japan. ^9^Department of Breast Surgery, Hiroshima City Hiroshima Citizens Hospital, Hiroshima, Japan. ^10^Division of Breast Oncology, Saitama Cancer Center, Saitama, Japan. ^11^Department of Breast and Endocrine Surgery, Osaka International Cancer Institute, Osako, Japan. ^12^Department of Breast Surgery, NHO Hokkaido Cancer Center, Sapporo, Japan, ^13^Department of Medical Oncology, Fukushima Medical University Hospital, Fukushima, Japan. ^14^Department of Breast Oncology, National Hospital Organization Shikoku Cancer Center, Ehime, Japan. ^15^Department of Breast Surgery, Kyoto University Hospital, Kyoto, Japan. ^16^Department of Biomedical Statistics and Bioinformatics, Kyoto University Graduate School of Medicine, Kyoto, Japan. ^17^Department of Diagnostic Pathology, Kyoto University Hospital, Kyoto, Japan. ^18^Breast Oncology Center, The Cancer Institute Hospital of JFCR, Tokyo, Japan. ^19^Department of Breast Surgery, Kyoto University Graduate school of medicine, Kyoto, Japan

**Additional File 1: Figures**


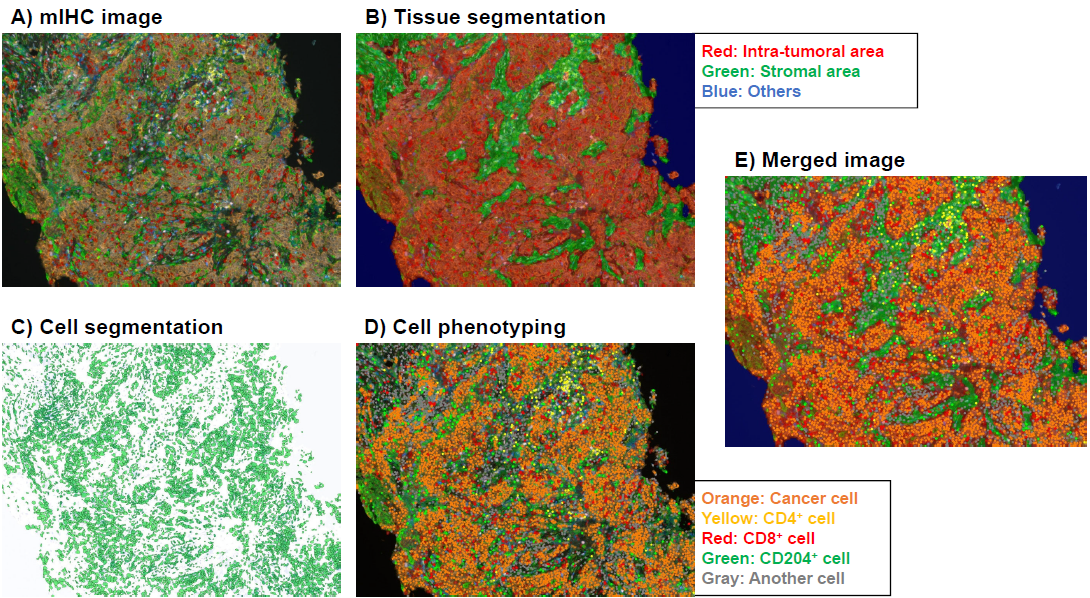


**Figure S1. Image analysis**

**Figure S2. Immune cell density according to pathological response in each treatment group: group A1 (A), group A2 (B), group B1 (C), group B2 (D)**

**Figure S3. Immune phenotype and pCR**
